# 2,3-Diphenyl­quinoxalin-1-ium chloride

**DOI:** 10.1107/S1600536810023883

**Published:** 2010-06-26

**Authors:** Wen-Sheng Wu

**Affiliations:** a School of Chemistry and Chemical Engineering, Zhaoqing University, Zhaoqing 526061, People’s Republic of China

## Abstract

The title compound, C_20_H_15_N_2_^+^·Cl^−^, was prepared by the reaction of benzil with *o*-phenyl­enediamine in refluxing ethanol and then crystallized in 5% hydro­chloric acid. The two phenyl rings are oriented at dihedral angles of 50.93 (8) and 50.28 (8)° with respect to the quinoxalin-1-ium ring system. The dihedral angle between the two phenyl rings is 56.71 (10)°. In the crystal, the cations and anions are linked by N—H⋯Cl and C—H⋯Cl inter­actions, forming chains along the *b* axis.

## Related literature

For general background to quinoxaline derivatives, see: Brock *et al.* (1999 ▶); Dailey *et al.* (2001 ▶); Page *et al.* (1998 ▶); Pascal *et al.* (1993 ▶). For a related structure, see: Wu *et al.* (2002 ▶).
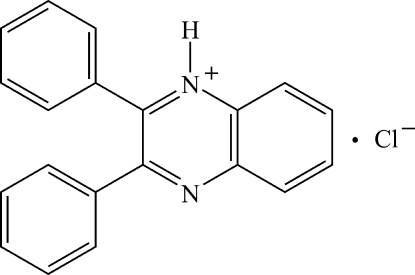


## Experimental

### 

#### Crystal data

C_20_H_15_N_2_^+^·Cl^−^
*M* *_r_* = 318.79
Monoclinic, 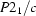

*a* = 10.498 (3) Å
*b* = 14.773 (5) Å
*c* = 11.359 (4) Å
β = 112.692 (3)°
*V* = 1625.3 (9) Å^3^
*Z* = 4
Mo *K*α radiation
μ = 0.24 mm^−1^
*T* = 293 K
0.25 × 0.22 × 0.20 mm

#### Data collection

Bruker SMART CCD area-detector diffractometer
Absorption correction: multi-scan (*SADABS*; Bruker, 2002 ▶) *T*_min_ = 0.943, *T*_max_ = 0.954
9558 measured reflections
2893 independent reflections
2257 reflections with *I* > 2σ(*I*)
*R*_int_ = 0.034

#### Refinement

*R*[*F* ^2^ > 2σ(*F* ^2^)] = 0.039
*wR*(*F* ^2^) = 0.109
*S* = 1.06
2893 reflections
209 parameters
H-atom parameters constrained
Δρ_max_ = 0.18 e Å^−3^
Δρ_min_ = −0.24 e Å^−3^

### 

Data collection: *SMART* (Bruker, 2002 ▶); cell refinement: *SAINT* (Bruker, 2002 ▶); data reduction: *SAINT*; program(s) used to solve structure: *SHELXS97* (Sheldrick, 2008 ▶); program(s) used to refine structure: *SHELXL97* (Sheldrick, 2008 ▶); molecular graphics: *ORTEP-3 for Windows* (Farrugia, 1997 ▶); software used to prepare material for publication: *WinGX* (Farrugia, 1999 ▶).

## Supplementary Material

Crystal structure: contains datablocks global, I. DOI: 10.1107/S1600536810023883/ci5102sup1.cif

Structure factors: contains datablocks I. DOI: 10.1107/S1600536810023883/ci5102Isup2.hkl

Additional supplementary materials:  crystallographic information; 3D view; checkCIF report

## Figures and Tables

**Table 1:** Hydrogen-bond geometry (Å, °)

*D*—H⋯*A*	*D*—H	H⋯*A*	*D*⋯*A*	*D*—H⋯*A*
N1—H1*A*⋯Cl1^i^	0.86	2.14	2.9684 (16)	160
C18—H18⋯Cl1	0.93	2.73	3.568 (2)	150

Symmetry code: (i) 
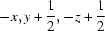
.

**Fig. 1.**
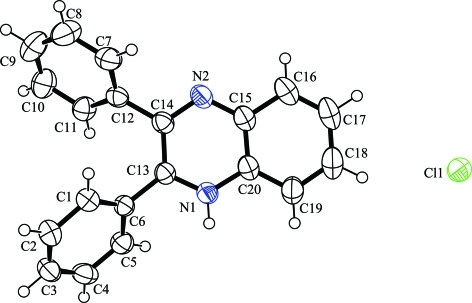
The asymmetric unit of the title compound, showing the atom-labelling scheme. Displacement ellipsoids are drawn at the 50% probability level.

## supplementary crystallographic information

### Comment

Quinoxaline and its derivatives have received considerable attention in the past several years due to their electronic properties (Page *et al.*, 1998), H-bonding ability (Pascal *et al.*, 1993), and their capacity to coordinate to metals and forming interesting three-dimensional structures (Wu *et al.*, 2002). Quinoxaline derivatives are also an important class of nitrogen containing heterocycles and constitute useful intermediates in organic synthesis which have been reported for their applications in the field of dyes (Brock *et al.*, 1999) and have also been used as building blocks for the synthesis of organic semiconductors (Dailey *et al.*, 2001). The title compound was synthesized as part of our study of these ligands.

The quinoxalin-1-ium ring system (N1/N2/C13-C20) is planar within ±0.035 (2) Å. The C1–C6 and C7–C12 phenyl rings form dihedral angles of 50.93 (8)° and 50.28 (8)°, respectively, with the quinoxalin-1-ium ring system. The dihedral angle between the two phenyl rings is 56.71 (10)°.

The N1—H1A···Cl1 and C18—H18···Cl1 hydrogen bonds are present in the crystal structure (Table 1), and these hydrogen bonds link the molecules into a chain along the *b* axis.

### Experimental

A 100 ml round-bottomed flask was charged with benzil (10.5 g, 0.05 mol), *o*-phenylenediamine (5.4 g, 0.05 mol) and ethanol (50 ml). The mixture was refluxed, and the reaction was monitored by thin-layer chromatography until complete consumption of the starting materials (4 h). The resulting solution was concentrated to dryness under reduced pressure. The dark-brown crude product was dissolved in methanol (30 ml). The solution was filtered and the filtrate was dissolved in 5% hydrochloric acid (30 ml) and set aside for three weeks to obtain yellow crystals.

### Refinement

H atoms were placed in calculated positions and refined as riding, with C–H = 0.93 Å, N–H = 0.86 Å and *U*_iso_(H) = 1.2*U*_eq_(C,N).

### Crystal data

**Table:**

C_20_H_15_N_2_^+^·Cl^−^	*F*(000) = 664
*M**_r_* = 318.79	*D*_x_ = 1.303 Mg m^−^^3^
Monoclinic, *P*2_1_/*c*	Mo *K*α radiation, λ = 0.71073 Å
Hall symbol: -P 2ybc	Cell parameters from 2838 reflections
*a* = 10.498 (3) Å	θ = 2.1–25.1°
*b* = 14.773 (5) Å	µ = 0.24 mm^−^^1^
*c* = 11.359 (4) Å	*T* = 293 K
β = 112.692 (3)°	Block, yellow
*V* = 1625.3 (9) Å^3^	0.25 × 0.22 × 0.20 mm
*Z* = 4	

### Data collection

**Table:**

Bruker SMART CCD area-detector diffractometer	2893 independent reflections
Radiation source: fine-focus sealed tube	2257 reflections with *I* > 2σ(*I*)
graphite	*R*_int_ = 0.034
φ and ω scans	θ_max_ = 25.1°, θ_min_ = 2.1°
Absorption correction: multi-scan (*SADABS*; Bruker, 2002)	*h* = −12→12
*T*_min_ = 0.943, *T*_max_ = 0.954	*k* = −17→17
9558 measured reflections	*l* = −13→10

### Refinement

**Table:**

Refinement on *F*^2^	Secondary atom site location: difference Fourier map
Least-squares matrix: full	Hydrogen site location: inferred from neighbouring sites
*R*[*F*^2^ > 2σ(*F*^2^)] = 0.039	H-atom parameters constrained
*wR*(*F*^2^) = 0.109	*w* = 1/[σ^2^(*F*_o_^2^) + (0.0549*P*)^2^ + 0.2666*P*] where *P* = (*F*_o_^2^ + 2*F*_c_^2^)/3
*S* = 1.06	(Δ/σ)_max_ = 0.001
2893 reflections	Δρ_max_ = 0.18 e Å^−^^3^
209 parameters	Δρ_min_ = −0.24 e Å^−^^3^
0 restraints	Extinction correction: *SHELXL97* (Sheldrick, 2008), Fc^*^=kFc[1+0.001xFc^2^λ^3^/sin(2θ)]^-1/4^
Primary atom site location: structure-invariant direct methods	Extinction coefficient: 0.068 (4)

### Special details

**Table:**

Geometry. All e.s.d.'s (except the e.s.d. in the dihedral angle between two l.s. planes) are estimated using the full covariance matrix. The cell e.s.d.'s are taken into account individually in the estimation of e.s.d.'s in distances, angles and torsion angles; correlations between e.s.d.'s in cell parameters are only used when they are defined by crystal symmetry. An approximate (isotropic) treatment of cell e.s.d.'s is used for estimating e.s.d.'s involving l.s. planes.
Refinement. Refinement of *F*^2^ against ALL reflections. The weighted *R*-factor *wR* and goodness of fit *S* are based on *F*^2^, conventional *R*-factors *R* are based on *F*, with *F* set to zero for negative *F*^2^. The threshold expression of *F*^2^ > σ(*F*^2^) is used only for calculating *R*-factors(gt) *etc*. and is not relevant to the choice of reflections for refinement. *R*-factors based on *F*^2^ are statistically about twice as large as those based on *F*, and *R*- factors based on ALL data will be even larger.

### Fractional atomic coordinates and isotropic or equivalent isotropic displacement parameters (Å^2^)

**Table:**

	*x*	*y*	*z*	*U*_iso_*/*U*_eq_	
Cl1	−0.00715 (5)	0.21487 (3)	0.19706 (5)	0.0593 (2)	
C13	0.25287 (17)	0.73467 (11)	0.12361 (16)	0.0396 (4)	
C6	0.23732 (18)	0.83023 (11)	0.15477 (17)	0.0410 (4)	
C14	0.35118 (18)	0.70084 (11)	0.07464 (17)	0.0420 (4)	
C1	0.35195 (19)	0.88047 (12)	0.22987 (19)	0.0497 (5)	
H1	0.4398	0.8553	0.2571	0.060*	
C12	0.44507 (19)	0.76230 (12)	0.04193 (17)	0.0442 (4)	
C20	0.18022 (19)	0.58334 (11)	0.13075 (17)	0.0456 (4)	
C8	0.6742 (2)	0.79882 (16)	0.0578 (2)	0.0698 (6)	
H8	0.7683	0.7863	0.0901	0.084*	
C5	0.10701 (19)	0.86889 (12)	0.11425 (18)	0.0483 (5)	
H5	0.0297	0.8355	0.0649	0.058*	
C2	0.3345 (2)	0.96830 (13)	0.2638 (2)	0.0593 (5)	
H2	0.4108	1.0016	0.3154	0.071*	
C15	0.2795 (2)	0.55363 (12)	0.08495 (17)	0.0483 (5)	
C4	0.0922 (2)	0.95729 (13)	0.14748 (19)	0.0544 (5)	
H4	0.0048	0.9834	0.1190	0.065*	
C3	0.2050 (2)	1.00672 (13)	0.2220 (2)	0.0586 (6)	
H3	0.1941	1.0660	0.2442	0.070*	
C18	0.1142 (2)	0.43199 (14)	0.1498 (2)	0.0664 (6)	
H18	0.0618	0.3905	0.1734	0.080*	
C7	0.5854 (2)	0.74393 (14)	0.0890 (2)	0.0578 (5)	
H7	0.6200	0.6942	0.1421	0.069*	
C17	0.2095 (3)	0.40062 (13)	0.1005 (2)	0.0677 (6)	
H17	0.2171	0.3388	0.0895	0.081*	
C11	0.3943 (2)	0.83694 (13)	−0.03710 (18)	0.0508 (5)	
H11	0.3006	0.8505	−0.0680	0.061*	
C19	0.0969 (2)	0.52241 (13)	0.16366 (19)	0.0564 (5)	
H19	0.0317	0.5430	0.1941	0.068*	
C16	0.2915 (2)	0.45917 (13)	0.0684 (2)	0.0604 (6)	
H16	0.3545	0.4374	0.0360	0.072*	
C10	0.4835 (2)	0.89082 (15)	−0.0695 (2)	0.0642 (6)	
H10	0.4493	0.9398	−0.1240	0.077*	
C9	0.6229 (2)	0.87214 (17)	−0.0213 (2)	0.0733 (7)	
H9	0.6827	0.9092	−0.0422	0.088*	
N1	0.17297 (15)	0.67478 (9)	0.14856 (14)	0.0431 (4)	
H1A	0.1134	0.6942	0.1774	0.052*	
N2	0.36430 (16)	0.61318 (10)	0.05822 (15)	0.0489 (4)	

### Atomic displacement parameters (Å^2^)

**Table:**

	*U*^11^	*U*^22^	*U*^33^	*U*^12^	*U*^13^	*U*^23^
Cl1	0.0709 (4)	0.0488 (3)	0.0721 (4)	−0.0091 (2)	0.0429 (3)	−0.0097 (2)
C13	0.0447 (9)	0.0376 (9)	0.0363 (10)	0.0010 (7)	0.0152 (8)	0.0010 (7)
C6	0.0522 (10)	0.0352 (9)	0.0420 (10)	0.0013 (8)	0.0251 (8)	0.0027 (8)
C14	0.0503 (10)	0.0391 (9)	0.0378 (10)	0.0026 (8)	0.0183 (8)	−0.0010 (7)
C1	0.0525 (11)	0.0440 (10)	0.0567 (12)	0.0004 (8)	0.0256 (9)	−0.0038 (9)
C12	0.0537 (11)	0.0414 (9)	0.0436 (11)	0.0014 (8)	0.0257 (9)	−0.0053 (8)
C20	0.0562 (11)	0.0367 (9)	0.0413 (10)	−0.0013 (8)	0.0162 (9)	−0.0007 (8)
C8	0.0539 (12)	0.0829 (16)	0.0802 (16)	0.0013 (11)	0.0342 (12)	0.0017 (13)
C5	0.0530 (11)	0.0476 (10)	0.0475 (11)	0.0025 (8)	0.0230 (9)	0.0024 (8)
C2	0.0686 (13)	0.0436 (10)	0.0722 (15)	−0.0085 (10)	0.0343 (12)	−0.0121 (10)
C15	0.0598 (11)	0.0384 (9)	0.0449 (11)	0.0005 (8)	0.0183 (9)	−0.0008 (8)
C4	0.0653 (13)	0.0503 (11)	0.0570 (12)	0.0179 (10)	0.0339 (11)	0.0089 (10)
C3	0.0823 (15)	0.0378 (10)	0.0691 (14)	0.0048 (10)	0.0441 (12)	−0.0011 (9)
C18	0.0847 (16)	0.0457 (11)	0.0668 (14)	−0.0142 (11)	0.0272 (12)	0.0036 (10)
C7	0.0577 (12)	0.0540 (11)	0.0684 (14)	0.0113 (10)	0.0317 (11)	0.0039 (10)
C17	0.0913 (16)	0.0349 (10)	0.0705 (15)	−0.0045 (11)	0.0242 (13)	−0.0022 (10)
C11	0.0544 (11)	0.0532 (11)	0.0456 (11)	−0.0004 (9)	0.0201 (9)	0.0025 (9)
C19	0.0684 (13)	0.0457 (11)	0.0567 (13)	−0.0071 (9)	0.0261 (10)	0.0020 (9)
C16	0.0754 (14)	0.0397 (10)	0.0624 (13)	0.0030 (10)	0.0225 (11)	−0.0066 (9)
C10	0.0742 (15)	0.0642 (13)	0.0553 (13)	−0.0057 (11)	0.0264 (11)	0.0136 (11)
C9	0.0718 (15)	0.0853 (16)	0.0735 (16)	−0.0172 (13)	0.0398 (13)	0.0062 (13)
N1	0.0508 (9)	0.0383 (8)	0.0444 (9)	0.0024 (7)	0.0230 (7)	0.0014 (7)
N2	0.0590 (9)	0.0388 (8)	0.0508 (10)	0.0033 (7)	0.0234 (8)	−0.0024 (7)

### Geometric parameters (Å, °)

**Table:**

C13—N1	1.323 (2)	C2—H2	0.93
C13—C14	1.438 (2)	C15—N2	1.367 (2)
C13—C6	1.480 (2)	C15—C16	1.420 (3)
C6—C5	1.387 (2)	C4—C3	1.371 (3)
C6—C1	1.391 (3)	C4—H4	0.93
C14—N2	1.323 (2)	C3—H3	0.93
C14—C12	1.489 (2)	C18—C19	1.365 (3)
C1—C2	1.386 (3)	C18—C17	1.400 (3)
C1—H1	0.93	C18—H18	0.93
C12—C7	1.387 (3)	C7—H7	0.93
C12—C11	1.392 (3)	C17—C16	1.366 (3)
C20—N1	1.372 (2)	C17—H17	0.93
C20—C19	1.402 (3)	C11—C10	1.383 (3)
C20—C15	1.403 (2)	C11—H11	0.93
C8—C9	1.377 (3)	C19—H19	0.93
C8—C7	1.381 (3)	C16—H16	0.93
C8—H8	0.93	C10—C9	1.379 (3)
C5—C4	1.385 (3)	C10—H10	0.93
C5—H5	0.93	C9—H9	0.93
C2—C3	1.378 (3)	N1—H1A	0.86
			
N1—C13—C14	117.35 (15)	C5—C4—H4	119.7
N1—C13—C6	116.70 (15)	C4—C3—C2	119.74 (18)
C14—C13—C6	125.88 (15)	C4—C3—H3	120.1
C5—C6—C1	119.53 (16)	C2—C3—H3	120.1
C5—C6—C13	119.95 (16)	C19—C18—C17	121.2 (2)
C1—C6—C13	120.44 (16)	C19—C18—H18	119.4
N2—C14—C13	121.60 (16)	C17—C18—H18	119.4
N2—C14—C12	116.54 (15)	C8—C7—C12	120.5 (2)
C13—C14—C12	121.85 (14)	C8—C7—H7	119.7
C2—C1—C6	119.62 (18)	C12—C7—H7	119.7
C2—C1—H1	120.2	C16—C17—C18	121.22 (19)
C6—C1—H1	120.2	C16—C17—H17	119.4
C7—C12—C11	119.33 (18)	C18—C17—H17	119.4
C7—C12—C14	119.44 (17)	C10—C11—C12	119.84 (18)
C11—C12—C14	121.22 (16)	C10—C11—H11	120.1
N1—C20—C19	121.12 (17)	C12—C11—H11	120.1
N1—C20—C15	117.01 (15)	C18—C19—C20	118.2 (2)
C19—C20—C15	121.79 (16)	C18—C19—H19	120.9
C9—C8—C7	119.8 (2)	C20—C19—H19	120.9
C9—C8—H8	120.1	C17—C16—C15	119.3 (2)
C7—C8—H8	120.1	C17—C16—H16	120.3
C4—C5—C6	119.88 (18)	C15—C16—H16	120.3
C4—C5—H5	120.1	C9—C10—C11	120.2 (2)
C6—C5—H5	120.1	C9—C10—H10	119.9
C3—C2—C1	120.58 (19)	C11—C10—H10	119.9
C3—C2—H2	119.7	C8—C9—C10	120.3 (2)
C1—C2—H2	119.7	C8—C9—H9	119.9
N2—C15—C20	121.46 (16)	C10—C9—H9	119.9
N2—C15—C16	120.29 (18)	C13—N1—C20	123.43 (15)
C20—C15—C16	118.24 (17)	C13—N1—H1A	118.3
C3—C4—C5	120.63 (18)	C20—N1—H1A	118.3
C3—C4—H4	119.7	C14—N2—C15	119.11 (16)
			
N1—C13—C6—C5	−49.4 (2)	C9—C8—C7—C12	−0.4 (3)
C14—C13—C6—C5	133.74 (19)	C11—C12—C7—C8	−0.2 (3)
N1—C13—C6—C1	127.41 (18)	C14—C12—C7—C8	178.61 (19)
C14—C13—C6—C1	−49.4 (3)	C19—C18—C17—C16	2.1 (3)
N1—C13—C14—N2	−1.8 (3)	C7—C12—C11—C10	1.1 (3)
C6—C13—C14—N2	175.02 (17)	C14—C12—C11—C10	−177.61 (18)
N1—C13—C14—C12	179.30 (16)	C17—C18—C19—C20	−1.9 (3)
C6—C13—C14—C12	−3.9 (3)	N1—C20—C19—C18	−176.65 (19)
C5—C6—C1—C2	0.4 (3)	C15—C20—C19—C18	−0.2 (3)
C13—C6—C1—C2	−176.49 (17)	C18—C17—C16—C15	−0.2 (3)
N2—C14—C12—C7	−49.1 (2)	N2—C15—C16—C17	177.39 (19)
C13—C14—C12—C7	129.83 (19)	C20—C15—C16—C17	−1.8 (3)
N2—C14—C12—C11	129.61 (18)	C12—C11—C10—C9	−1.6 (3)
C13—C14—C12—C11	−51.4 (2)	C7—C8—C9—C10	−0.1 (4)
C1—C6—C5—C4	0.8 (3)	C11—C10—C9—C8	1.1 (4)
C13—C6—C5—C4	177.62 (16)	C14—C13—N1—C20	0.4 (2)
C6—C1—C2—C3	−1.2 (3)	C6—C13—N1—C20	−176.73 (16)
N1—C20—C15—N2	−0.6 (3)	C19—C20—N1—C13	177.36 (17)
C19—C20—C15—N2	−177.17 (17)	C15—C20—N1—C13	0.7 (3)
N1—C20—C15—C16	178.58 (17)	C13—C14—N2—C15	2.0 (3)
C19—C20—C15—C16	2.0 (3)	C12—C14—N2—C15	−179.07 (15)
C6—C5—C4—C3	−1.1 (3)	C20—C15—N2—C14	−0.8 (3)
C5—C4—C3—C2	0.3 (3)	C16—C15—N2—C14	−179.90 (18)
C1—C2—C3—C4	0.9 (3)		

### Hydrogen-bond geometry (Å, °)

**Table:**

*D*—H···*A*	*D*—H	H···*A*	*D*···*A*	*D*—H···*A*
N1—H1A···Cl1^i^	0.86	2.14	2.9684 (16)	160
C18—H18···Cl1	0.93	2.73	3.568 (2)	150

Symmetry codes: (i) −*x*, *y*+1/2, −*z*+1/2.
